# Prediction of the Network Pharmacology-Based Mechanism for Attenuation of Atherosclerosis in Apolipoprotein E Knockout Mice by *Panax notoginseng* Saponins

**DOI:** 10.1155/2020/8574702

**Published:** 2020-04-22

**Authors:** Linzi Long, Zikai Yu, Hua Qu, Ning Wang, Ming Guo, Xuezhong Zhou, Changgeng Fu, Zhuye Gao

**Affiliations:** ^1^Academy of Integrative Medicine, Fujian University of Traditional Chinese Medicine, Fuzhou 350112, China; ^2^Department of Geriatrics, Xiyuan Hospital, China Academy of Chinese Medical Sciences, Beijing 100091, China; ^3^Department of Cardiovascular Disease Center, Xiyuan Hospital, China Academy of Chinese Medical Sciences, Beijing 100091, China; ^4^School of Computer and Information Technology and Beijing Key Lab of Traffic Data Analysis and Mining, Beijing Jiaotong University, Beijing 100044, China

## Abstract

This study investigated whether *Panax notoginseng* saponins (PNS) reduced atherosclerotic lesion formation in apolipoprotein E knockout (ApoE-KO) mice and illustrated the potential mechanism for a network pharmacology approach. Pharmacodynamics studies on ApoE-KO mice with atherosclerosis (AS) showed that PNS generated an obvious anti-AS action. Then, we explored the possible mechanisms underlying its anti-AS effect using the network pharmacology approach. The main chemical components and their targets of PNS were collected from TCMSP public database and SymMap. The STRING v11.0 was used to establish the protein-protein interactions of PNS. Furthermore, the Gene Ontology (GO) function and KEGG pathways were analyzed using STRING to investigate the possible mechanisms involved in the anti-AS effect of PNS. The predicted results showed that 27 potential targets regulated by DSLHG were related to AS, including ACTA2, AKT1, BCL2, and BDNF. Mechanistically, the anti-AS effect of PNS was exerted by interfering with multiple signaling pathways, such as AGE-RAGE signaling pathway, fluid shear stress and atherosclerosis, and TNF signaling pathway. Network analysis showed that PNS could generate the anti-AS action by affecting multiple targets and multiple pathways and provides a novel basis to clarify the mechanisms of anti-AS of PNS.

## 1. Introduction

Atherosclerosis (AS) is a multifactorial disease that develops over many years, with clinical symptoms becoming obvious in the late stages of many diseases. Inflammation [1] and decompensation of lipid metabolism [2] are associated with the pathogenesis of AS. The results of population studies suggest that adopting traditional Chinese medicine (TCM) could protect against cardiovascular disease [3–5]. *Panax notoginseng* saponins (PNS) are one of the most important compounds stemming from the roots of the *Panax notoginseng,* which has been traditionally used as a blood-supplementing and hemostatic medicine in China for thousands of years.

**Figure 1:**
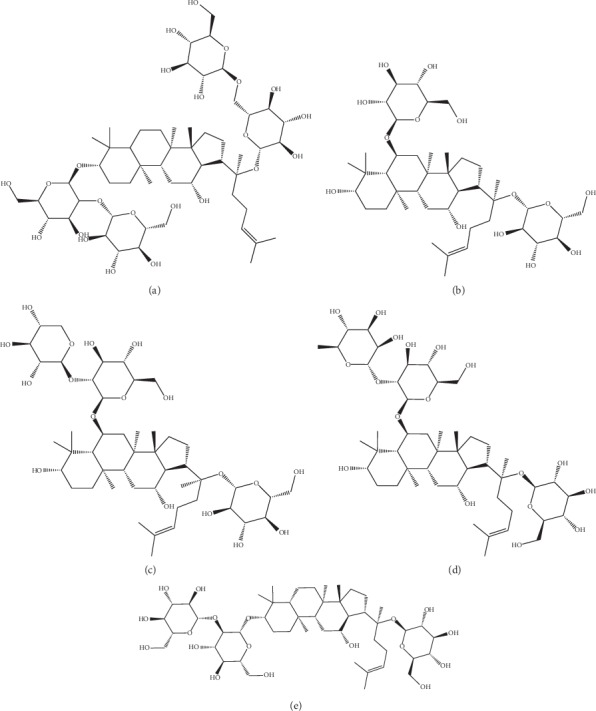
Main compound structure of *Panax notoginseng* saponins: (a) ginsenoside Rb1; (b) ginsenoside Rg1; (c) notoginsenoside R1; (d) ginsenoside Re; (e) ginsenoside Rd.

To date, at least twenty-seven saponins in PNS have been identified and notoginsenoside R1, ginsenoside Rb1, ginsenoside Rg1, ginsenoside Re, and ginsenoside Rd (structure in Figure 1) are the major effective constituents and have been the topic of much research in the area of cardiovascular disease [6]. Previous studies have indicated that PNS may ameliorate myocardial ischemia injury by decreasing oxidative stress and repressing the inflammatory cascade [7]. Another study demonstrated that PNS attenuated the injury of human umbilical vascular endothelial cells (HUVECs) induced by oxidized low-density lipoprotein (ox-LDL) [8]. ApoE is an important ligand for the uptake of lipoproteins by many receptors in the LDLR gene family, and deficiency of ApoE leads to the accumulation of cholesterol ester-enriched particles [9]. ApoE-KO mice develop severe atherosclerosis on a fat-containing diet, shortly became a powerful tool in atherosclerosis research [10]. Given the concern about the bioavailability of PNS *in vivo*, we examined if PNS prevented or reduced the formation of atherosclerotic lesions in ApoE-KO mice, investigated the mechanisms by which PNS exerted its anti-AS effects, and built a network of interactions among related targets. Moreover, we conducted GO function analysis and relevant pathway enrichment analysis for the potential mechanism.

## 2. Methods

### 2.1. Drugs and Antibodies

PNS were purchased from Kunming Pharmaceutical Corporation (KPC) Pharmaceuticals, Inc. (Item. no. SKQ2017001; Kunming Yunnan Province, China). Notoginsenoside R1 (percentage: 9.8%; PubChem CID: 441934), ginsenoside Rb1 (percentage: 32.1%; PubChem CID: 9898279), ginsenoside Rg1 (percentage: 30.8%; PubChem CID: 441923), ginsenoside Re (percentage: 4.3%; PubChem CID: 441921), and ginsenoside Rd (percentage: 8.3%; PubChem CID: 11679800) are the major effective constituents (Figure 1). The total concentration of these main constituents is 85.3% (Supplementary Materials). Simvastatin (Zocor; 20 mg/pill) was purchased from Merck Pharmaceutical Co., Ltd. (Hangzhou, Zhejiang Province, China). Goat anti-rabbit IgG H&L (Item. no. ab6721) was purchased from Abcam (Cambridge, MA, USA). The secondary antibodies used were part of a general-purpose two-step immunohistochemical kit (Item. no. PV. 6000; ZSGB Biological Technology; OriGene Technologies, Inc., Rockville, MD, USA). The DAB kit was also purchased from ZSGB Biological Technology. The mouse IL-1*β* ELISA Kit (Item. no. EM001-48) was purchased from ExCell (Shanghai, China). The mouse matrix metalloproteinase MMP-9, ELISA kit (Item. no. MU30613), and mouse tissue inhibitors of metalloproteinase-1, and the TIMP-1 ELISA Kit (Item. No. MU30070) were purchased from BiosWamp (Beijing, China). Oil red O solution was purchased from Sigma Chemical (St Louis, MO, USA).

### 2.2. Animal Grouping and Treatment

The present study was approved by the Animal Care and Use Committee of Xiyuan Hospital of the China Academy of Chinese Medical Sciences (Beijing, China). A total of 15 male apolipoprotein E knockout (ApoE-KO) mice and 3 male wild-type mice (strain: C57BL/6J; weight: 22 ± 2.5 g; age: 8 weeks) were purchased from Changzhou Cavens Bioscience Co., Ltd. (Changzhou, Jiangsu, China). The mice were housed in humidity-controlled rooms (60 ± 10%) at 24 ± 1°C with a 12 h light/dark cycle. After a 7-day adaptation period, fifteen ApoE-KO mice were fed with an atherogenic high-fat diet (HFD; normal diet supplemented with 0.5% cholesterol, 10% yolk powder, and 5% pork lard) for 12 weeks. After that, these animals were randomized to receive simvastatin (20 mg/kg/d, *n* = 5), PNS (60 mg/kg/d (according to the literature [11] and preliminary experimental results), *n* = 5), or no drug (*n* = 5) for 8 weeks. The mice received the HFD until the end of the study. The C57BL/6J male mice were used as a control group (*n* = 3) and received a normal chow diet. Both diet and water were available ad libitum. Amounts of PNS were prepared as previously described [12]. At the end of the experiment, animals were fasted overnight and sacrificed. Blood samples were collected by removing the eyeball, and tissue samples from the aortic valve were obtained.

### 2.3. Histopathological Analysis

Parts of the aortic root were fixed in 10% buffered formalin at room temperature for 48 h, embedded in paraffin, and sliced into 3 *μ*m thick sections. Sections were processed for hematoxylin-eosin (H&E) staining and oil red O staining. Histological images were recorded with an Olympus (CX31) microscope. Quantitative morphometric analysis was conducted using Image Pro Plus (version 4.5) to determine plaque area and intima-media thickness (IMT).

### 2.4. Serum Lipid Measurement and Enzyme-Linked Immunosorbent Assay (ELISA) Test

Blood samples were left at room temperature for 30 min and then centrifuged at 1000 ×g for 10 min at 4°C prior to storage at −70°C for later biochemical analysis. Blood was obtained to determine total cholesterol (TC), triacylglycerides (TG), LDL-C, and high-density lipoprotein cholesterol (HDL-C) levels using a Roche Cobas 800 automatic biochemical analyzer (Roche Diagnostics GmbH, Mannheim, Germany). Levels of interleukin-1 beta (IL-1*β*), matrix metallopeptidase 9 (MMP-9), and tissue inhibitor of metalloproteinase-1 (TIMP-1) were measured using commercially available assay kits according to the manufacturer's protocol.

### 2.5. Network Pharmacology Analyses

The main chemical components and their targets of PNS were obtained from a Traditional Chinese Medicine Systems Pharmacology Database (TCMSP) and Analysis Platform (http://lsp.nwu.edu.cn/tcmsp.php) and SymMap (http://www.symmap.org/). The protein-protein interaction network database (STRING v11.0, https://string-db.org/) was used to establish the protein-protein interactions of PNS and generate a visualization figure. Then, the Gene Ontology (GO) function and KEGG pathways of 32 common targets were analyzed using the STRING resource.

### 2.6. Statistical Analysis

Statistical analysis was performed using SPSS version 20.0 (SPSS Inc., Chicago).

One-way analysis of variance with a post hoc Bonferroni test was used to compare treatments. Data were expressed as the mean ± standard deviation, and *P* < 0.05 was considered to indicate a statistically significant difference.

## 3. Results

### 3.1. Animal Experiments

#### 3.1.1. Effect of PNS on Atherosclerotic Lesions

**Figure 2:**
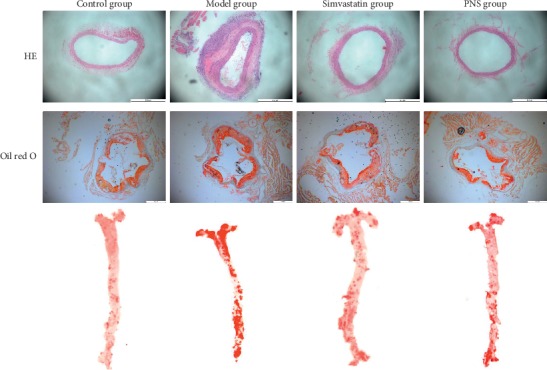
Effect of PNS on atherosclerotic lesion. Atherosclerotic lesion pathological changes in each group were observed using light microscopy (magnification, ×100) (*N* = 3–5).

**Figure 3:**
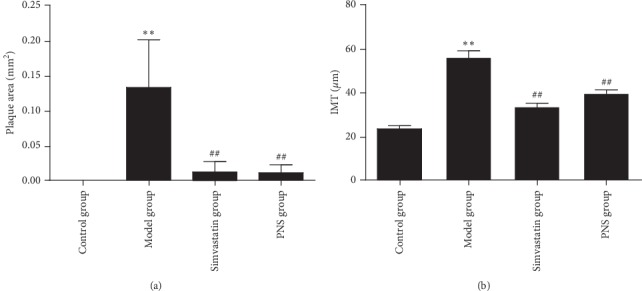
Effect of PNS on plaque area and IMT. Notes: data are expressed as the mean ± standard deviation (model, simvastatin, and PNS groups; control group). ^*∗∗*^*P* < 0.01 vs. the control group; ^##^*P* < 0.01 vs. the model group (*N* = 3–5).

As demonstrated in Figure 2, atherosclerotic changes were evaluated using H&E and oil red O staining. Compared with the model (untreated) group, PNS and simvastatin treatments ameliorated atherosclerotic lesions in ApoE-KO mice. The plaque area and IMT in the untreated group increased significantly compared with the control group (*P* < 0.01, Figure 3). In the PNS and simvastatin groups, the plaque area and IMT were significantly decreased compared with the untreated group (*P* < 0.01, Figure 3). These results demonstrated that PNS inhibited plaque area, IMT, and lipid deposition in atherosclerotic lesions.

#### 3.1.2. Effect of PNS on Serum Lipids

**Figure 4:**
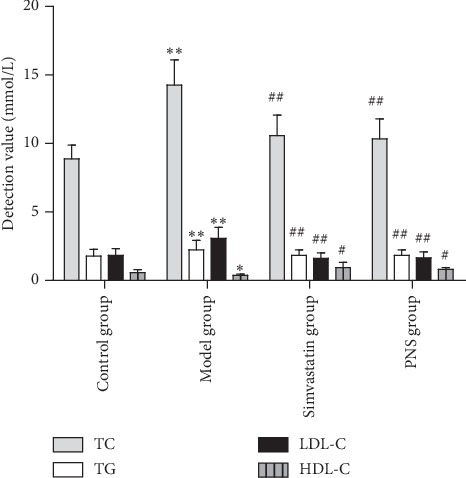
Effect of PNS on serum lipid. Notes: data are expressed as the mean ± standard deviation (model, simvastatin, and PNS groups; control group). ^*∗∗*^*P* < 0.01 vs. the control group; ^##^*P* < 0.01 vs. the model group; ^#^*P* < 0.05 vs. the model group (*N* = 3–5).

As demonstrated in Figure 4, TC, TG, and LDL-C levels in the untreated group increased significantly compared with the control group (*P* < 0.01). Compared with the untreated group, the PNS and simvastatin groups exhibited significantly decreased levels of TC, TG, and LDL-C (all *P* < 0.01). Furthermore, levels of HDL-C in the untreated group decreased significantly compared with the control group (*P* < 0.05; Figure 4). Compared with the untreated group, PNS and simvastatin significantly increased the HDL-C levels (both *P* < 0.05). There was no significant difference between simvastatin and PNS. These results indicated that PNS ameliorated atherogenic HFD-induced changes in TC, TG, LDL-C, and HDL-C levels.

#### 3.1.3. Effect of PNS on Levels of MMP-9, TIMP-1, and IL-1*β*

**Figure 5:**
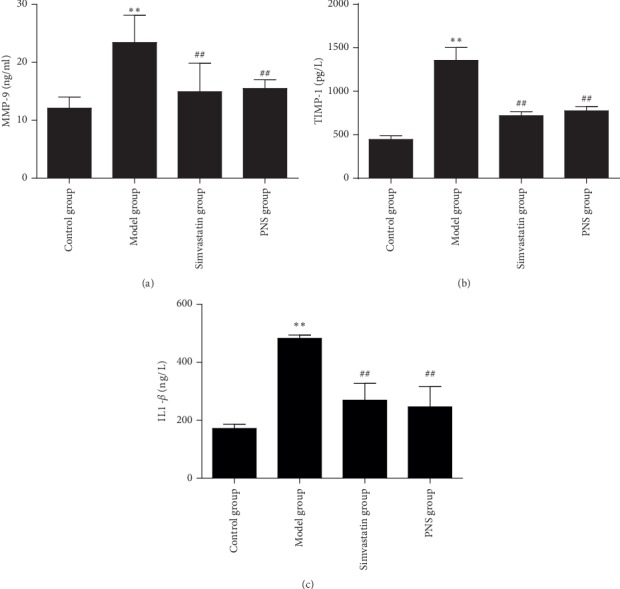
Effect of PNS on levels of MMP-9, TIMP-1, and IL-1*β*. Data are expressed as the mean ± standard deviation (model, simvastatin, and PNS groups; control group). ^*∗∗*^*P* < 0.01 vs. the control group; ^##^*P* < 0.01 vs. the model group (*N* = 3–5).

The plaque fibrous cap factors, MMP-9 and TIMP-1, seem to play critical roles in the stages of plaque rupture and erosion [13]. As demonstrated in Figure 5, MMP-9, TIMP-1, and IL-1*β* levels in the untreated group increased significantly compared with the control group (*P* < 0.01). Compared with the untreated group, the PNS and simvastatin groups exhibited significantly decreased levels of MMP-9, TIMP-1, and IL-1*β* (all *P* < 0.01). There was no significant difference between simvastatin and PNS. These data indicated that PNS could have an anti-inflammatory role and regulate the plaque fibrous cap factors.

### 3.2. Network Pharmacology Results

#### 3.2.1. Compounds-Targets-Disease and PPI Network Construction

**Figure 6:**
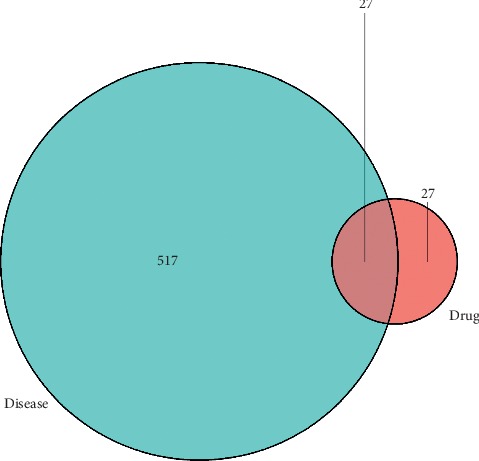
The compounds-targets-disease network.

**Figure 7:**
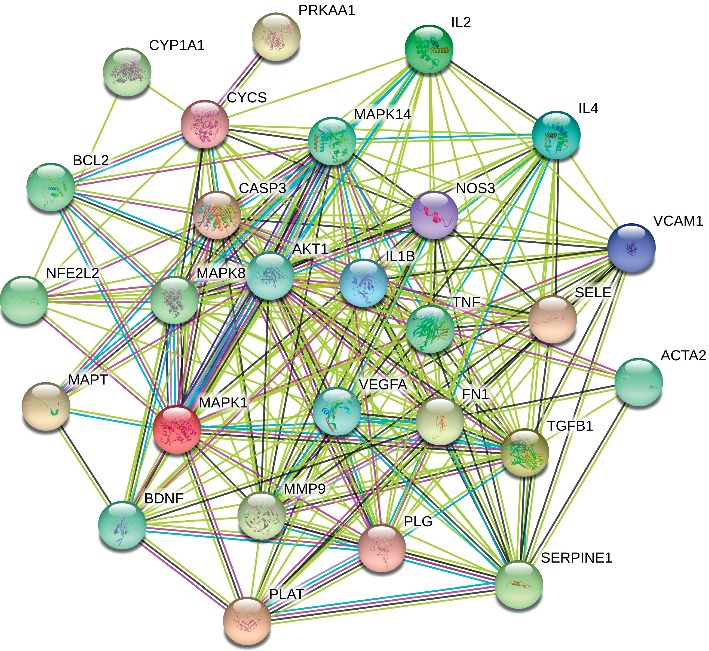
The protein interaction of the overlap of the potential targets.

As shown in Figure 6, based on the analysis of the network, six ingredients including notoginsenoside R1, ginsenoside Rg1, ginsenoside Rb1, ginsenoside Rd, ginsenoside Re, and ginsenoside Rg2, associated with 54 drug targets, were selected. Totally, this compounds-targets-disease network is composed of 54 active drugs and 517 disease targets. Then, 27 target genes associated with active ingredients and AS were imported into the STRING database for PPI network construction (Figure 7).

#### 3.2.2. Gene Ontology and Kyoto Encyclopedia of Genes and Genomes Signaling Pathway Analysis

**Figure 8:**
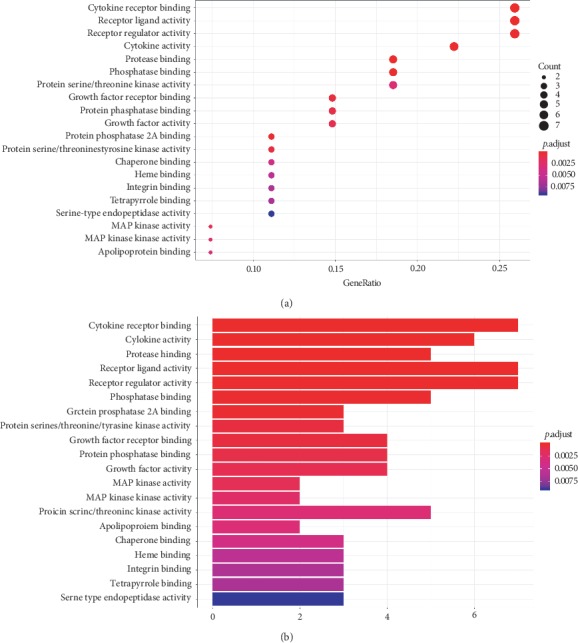
GO function analysis. (a) Bubble chart. (b) Bar graph.

**Figure 9:**
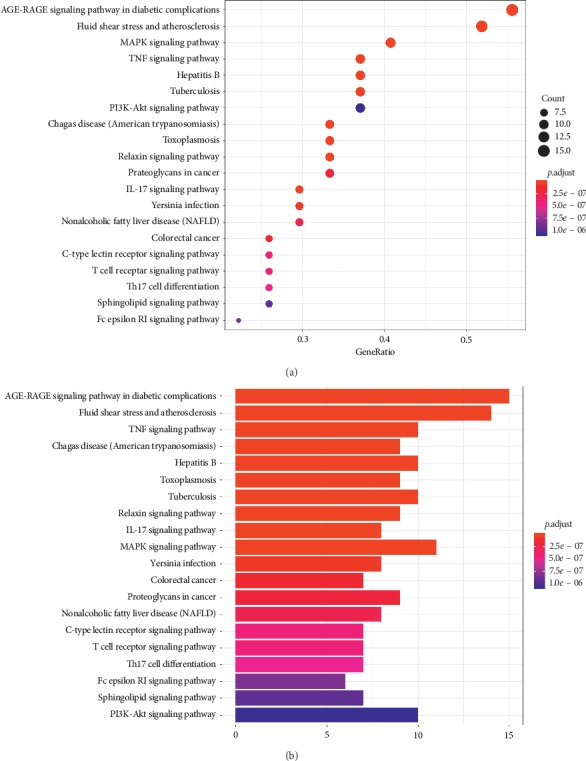
KEGG pathway enrichment analysis for core targets of PNS acting on AS. (a) Bubble chart. (b) Bar graph.

Furthermore, Gene Ontology (GO) analysis of the 27 targets was performed. The results of GO analysis of the predicted key targets of PNS are shown in Figure 8, which lists the GO terms with low *P* values and increased target enrichment. The results showed that these targets were mainly related to physiological mechanisms, such as cytokine receptor binding, cytokine activity, and protease binding. To determine the relevant signaling pathways, we conducted pathway enrichment analysis using KEGG pathways. A total of 126 KEGG signaling pathways were significantly enriched (*P* < 0.05). The top 10 pathways with lower *P* values and increased gene enrichment are listed in Figure 9 and include AGE-RAGE signaling pathway, fluid shear stress and atherosclerosis, and the TNF signaling pathway. The results indicated multiple channels and mechanisms of PNS against AS.

## 4. Discussion

Our study showed that PNS significantly attenuated AS lesion formation, decreased blood lipids, and inhibited inflammatory factors in ApoE-KO mice. In conclusion, PNS inhibited the development of atherosclerosis. On this basis, we explored the possible mechanisms underlying its anti-AS effect using the network pharmacology approach.

Simvastatin is a lipid-lowering agent used to treat hypercholesterolemia and to reduce the risk of heart disease. Studies [14, 15] reported that simvastatin inhibits AS in mice mainly through the inhibition of cholesterol syntheses. In the present study, the plaque formation, IMT, and lipid content in the aortas of ApoE-KO mice were promoted by an HFD. PNS treatment, similar to simvastatin, attenuated atherosclerotic lesions, which was demonstrated by the significant reduction in the atherosclerotic plaque area and IMT. Numerous studies have established the essential relationship between hyperlipidemia and AS for more than a century [16, 17]. High levels of TC, TG, and LDL-C are thought to contribute to the development of atherogenic properties [18]. Currently, studies have demonstrated that AS could be markedly alleviated by reducing TC, TG, and LDL-C levels [19, 20]. In addition, increased expression of the main HDL protein might mediate protection from AS [21, 22]. Therefore, low levels of TC, TG, and LDL-C and high levels of HDL would ameliorate AS. Previous studies have reported that PNS have been reported to prevent AS by regulating lipid profiles [23]. The results of the present study are consistent with previous studies, indicating that PNS reduced plaque formation and lipid modulation.

As a family of zinc-dependent endopeptidases, matrix metalloproteinases (MMPs) are responsible for tissue remodeling by cleaving all structural elements of the extracellular matrix (ECM) and activating cytokines [24]. Clinical research showed that serum MMP-9 levels could be an early marker of plaque rupture contributing to acute coronary syndrome [25]. The pathological plaque rupture process that involves inflammation and fibrosis in AS could be regulated by MMP-9 and TIMP-1 [26]. In the present study, the expression of MMP-9 and TIMP-1 was regulated by PNS, illuminating plaque vulnerability to its products. Over the last two decades, growing evidence has revealed the main mechanism in the pathogenesis of AS, a chronic inflammatory disease [27], is inflammation [28, 29]. Cytokines are known to have pivotal roles in the complex inflammatory response involved in all steps of the formation of an atherosclerotic plaque [30]. IL-1*β*, one of the proatherogenic cytokines, promotes the migration of macrophages and influences almost all cells involved in AS [31]. Experimental studies have shown that proatherogenic cytokine knockout mice showed decreased atherogenesis [32, 33]. The present study revealed that PNS attenuated the expression of IL-1*β*, suggesting that PNS played an anti-inflammatory role in AS.

Through the analysis of a PNS compound-target-pathway network, we found that the main components of PNS could act on multiple pathways. Vascular endothelial cells are exposed to fluid shear stress, which modulates vascular pathophysiology. It has been suggested that regions of low flow magnitude, various metrics of flow disturbance, and the flow direction have the best correlation with atherosclerosis [34].

Our finding that PNS could regulate fluid shear stress and the atherosclerosis pathway is consistent with these results. These will lay the foundation for improving the clinical applications of blood circulation-activating decoctions and can help provide strategies and methods for clinical studies of TCM in atherosclerotic treatment.

## 5. Conclusion

PNS could attenuate atherosclerotic lesions. The network pharmacology-predicted signaling pathways provide ideas for experiments to verify the key mechanisms of the anti-AS effects of PNS in the future. Thus, this study enhanced the therapeutic potential of PNS in atherosclerotic disease.

## Data Availability

The data used to support the findings of this study are included within the article and the supplementary information files.

## Supplementary Materials

Supplementary Materials Supplementary 1: certificate of Chromatogram and Analysis.
