# Supplementary material for: Prediction of the Network Pharmacology-Based Mechanism for Attenuation of Atherosclerosis in Apolipoprotein E Knockout Mice by Panax notoginseng Saponins
Source: Evid Based Complement Alternat Med. 2020 Apr 22;2020:8574702. doi: 10.1155/2020/8574702 (PMC7193284; doi:10.1155/2020/8574702)
Supplement: Supplementary Materials — Supplementary 1: certificate of Chromatogram and Analysis. [file 8574702.f1.pdf]

Chromatogram

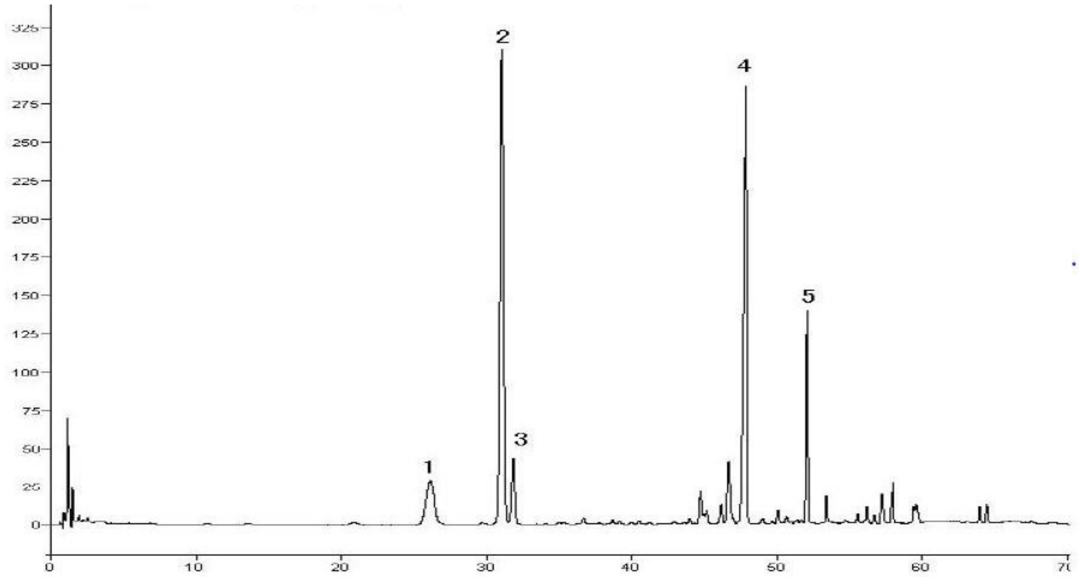

1： *Notoginsenoside R*1　　2： *ginsenoside Rg1*　　3： *ginsenoside Re*

4： *ginsenoside Rb1*　　5： *ginsenoside Rd*

Certificate of Analysis

检验项目 Test items	标 准 规 定 Specifications	检 验 结 果 Results	单项判定 Individual Conclusion
重量差异 Variation	±10%	±10%	合格 Compliant
崩解时限 Disintegration	应≤60 分钟 Not more than 60 minutes	6 分钟 6 minutes	合格 Compliant
微生物限度 Microbial limit	需氧菌总数 Bacteria:≤10^3cfu/g	<10 cfu/g	合格 Compliant
	霉菌和酵母菌总数 Molds and yeasts: ≤10^2cfu/g	<10 cfu/g	合格 Compliant
	大肠埃希菌 Escherichia Coli：1g 中不得检出 none in 1g	符合规定 Compliant	合格 Compliant
指纹图谱 Fingerprint	供试品与对照品指纹图谱经相似度计算，其相似度不得低于 0.95 The similarity between the tested product and the reference product should not be less than 0.95	1.000	合格 Compliant
【含　量】Assay（标示量计 Labeled amount）	三七皂苷 Notoginsengnoside R_1:≥5.0%	9.8%	合格 Compliant
	人参皂苷 Ginsengnoside Rg_1： ≥25.0%	30.8%	合格 Compliant
	人参皂苷 Ginsengnoside Re： ≥2.5%	4.3%	合格 Compliant
	人参皂苷 Ginsengnoside Rb_1:≥27.0%	32.1%	合格 Compliant
	人参皂苷 Ginsengnoside Rd:≥5.0%	8.3%	合格 Compliant
	R_1+Rg_1+ Re + Rb_1+ Rd:≥75%	85%	合格 Compliant
